# Are orthopaedic randomized controlled trials as statistically fragile as portrayed? A call for improved interpretation of the statistical fragility index

**DOI:** 10.1002/jeo2.12042

**Published:** 2024-05-31

**Authors:** Jacob F. Oeding, Olufemi R. Ayeni, Eric Hamrin Senorski, Stefano Zaffagnini, Alberto Grassi, Kristian Samuelsson

**Affiliations:** ^1^ Mayo Clinic Alix School of Medicine, Mayo Clinic Rochester Minnesota USA; ^2^ Department of Orthopaedics, Institute of Clinical Sciences, The Sahlgrenska Academy University of Gothenburg Gothenburg Sweden; ^3^ Division of Orthopaedics, Department of Surgery McMaster University Hamilton Ontario Canada; ^4^ Department of Health and Rehabilitation, Institute of Neuroscience and Physiology, The Sahlgrenska Academy University of Gothenburg Gothenburg Sweden; ^5^ IIa Clinica Ortopedica e Traumatologica, IRCCS Istituto Ortopedico Rizzoli Bologna Italy

**Abbreviations**
FI: fragility index
RCT: randomized controlled trial

The fragility index (FI) was first proposed by Walsh et al. in 2014 as a statistical tool to augment *p* values and 95% confidence intervals and to facilitate the identification of trials with less robust results [10]. Specifically, in the context of increased criticism around threshold p‐values as an overly simple concept to determine whether a treatment effect is truly present, the FI determines the minimum number of patients in a trial who would need to experience an alternative or ‘flipped’ outcome (such as a patient experiencing treatment *success* actually experiencing treatment *failure*) to reverse the significance of that trial. Calculation of the FI entails *simultaneously* adding and subtracting outcome events (i.e., treatment successes) and nonevents (i.e., treatment failures) in a sequential manner until the p‐value of that trial rises above the commonly applied 0.05 threshold level, thus providing a measure of the trials' ‘fragility’ or ‘robustness’ to alternate patient outcomes [10].

Recently, the orthopaedic literature has experienced a surge in the number of publications applying the FI to study the fragility of randomized controlled trials (RCTs) in orthopaedics, with FI‐based systematic reviews published across virtually all subspecialties [2, 3, 4, 5, 6, 7, 8, 9]. Nevertheless, each of these seems to propose some form of the same conclusion: ‘RCTs published on [insert topic] are frequently fragile and their significance can be flipped by changes in the outcomes of only a handful of patients’. This is regardless of whether the median FI was found to be just one patient or seven patients. This raises the question: if every treatment, subspecialty, or topic in orthopaedic surgery is characterized by fragile RCTs, are there any conclusions in which we *can* have confidence? Or is it actually the case that without official thresholds as to what constitutes a ‘fragile’ versus ‘robust’ RCT, authors feel pressured to come up with definitive and attention‐grabbing conclusions regarding the statistical fragility of RCTs on their chosen topic, particularly in an era of ‘publish or perish’? This ‘gold‐rush’ for publications applying the FI has led to an abundance of studies that warn of RCT fragility, resulting in a positive feedback loop in which the latest FI‐based review uses the claims and methodology of the previous FI‐based review to support its own claims of trial fragility.

While Xin and Lin have proposed guidelines for interpreting FIs based on an empirical analysis of studies in the Cochrane Library [11], these have yet to be validated and are rarely referenced. Instead, authors have frequently defaulted to direct comparisons between the FI and the number of patients lost to follow‐up in an attempt to define a threshold by which an RCT can be considered ‘fragile’. Thus, it is common to read conclusions like: ‘Ten of the trials reported that the number of patients lost to follow‐up exceeded the fragility index, meaning that results of the patients lost to follow‐up could theoretically completely reverse the study conclusions’; or ‘These figures are cause for significant concern, as it is possible that in approximately two‐thirds of the RCTs, the significance could have been reversed had the trial had complete follow‐up’ or ‘Significant outcomes reported in orthopaedic surgery RCTs are often fragile and outcomes of the patients lost to follow‐up could alter the significance of the results and hence substantial caution is needed before translating their results into clinical practice’.

The problem with such comparisons is that the FI and the number of patients lost to follow‐up are fundamentally incomparable on a statistical level, despite the direct comparisons being made by a majority of FI‐based systematic reviews. This leads to the potential for what are erroneous claims that the results of a statistically significant RCT could be flipped simply by maintaining study follow‐up, as the statistical effect of a patient converting from one outcome group to the other may be drastically different from the effect of adding an additional patient to a specific outcome group. For example, take a theoretical RCT consisting of 100 patients (50 in each treatment arm), with 40 out of 50 patients in the experimental group experiencing treatment success and 26 out of 50 patients in the control group experiencing treatment success. This trial has a *p* value of 0.006 and an FI of 4, meaning that at least four patients included in the trial results would need to experience the opposite outcome for the trial to flip from significant to non‐significant. If there were equivalency of statistical impact between the number of patients lost to follow‐up and the FI, as the majority of FI‐based studies seem to claim, adding four patients lost to follow‐up back to the trial could theoretically result in a *p* value that exceeded 0.05. However, in practice, adding four patients to this trial in the distribution that maximizes the effect on the *p* value (maximally increases the *p* value) while assuming an equal chance that the patients lost to follow‐up were from the control group as were from the experimental group is only able to increase the *p* value to 0.023, failing to demonstrate a reversal of significance (Figure 1). Regardless of the outcomes these four additional patients experience, there is no statistically feasible way for them to reverse the significance of the trial. In fact, a reversal of significance would not be achieved until eight patients lost to follow‐up are added to the trial in the distribution of maximal significance reversal, assuming an equal distribution from experimental and control groups, which is twice the original trial's FI. Thus, comparisons between the number of patients lost to follow‐up and the FI, specifically those misinterpreting the number of patients lost to follow‐up as being equivalent to the FI, have resulted in an overabundance of claims of RCT fragility and perhaps unwarranted warnings of fragile conclusions around orthopaedic RCTs.

**Figure 1:**
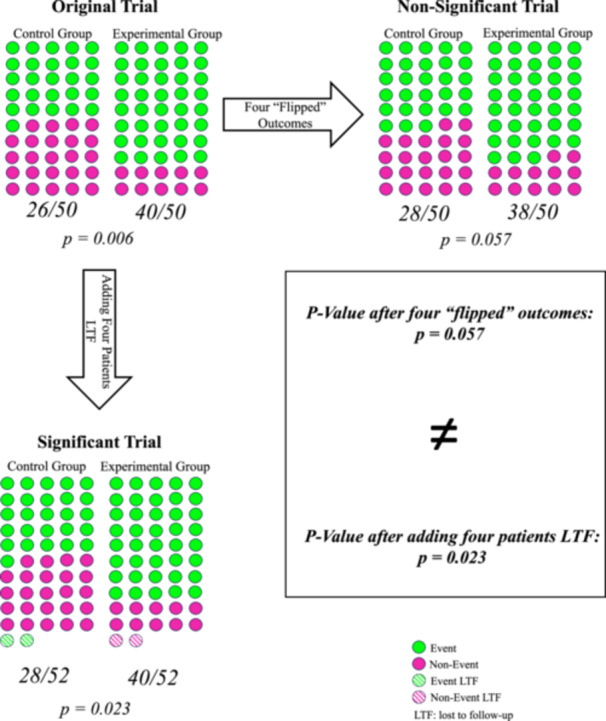
A theoretical RCT consisting of 100 patients (50 in each treatment arm), with 40 out of 50 patients in the experimental group experiencing treatment success and 26 out of 50 patients in the control group experiencing treatment success. This trial has a *p* value of 0.006 and an FI of 4, meaning that at least four patients included in the trial results would need to experience the opposite outcome for the trial to flip from significant to non‐significant. If there were equivalency of statistical impact between the number of patients lost to follow‐up and the FI, as the majority of FI‐based studies seem to claim, adding four patients lost to follow‐up back to the trial could theoretically result in a *p* value that exceeded 0.05. However, in practice, adding four patients to this trial in the distribution that maximizes the effect on the *p* value (maximally increases the *p* value) while assuming an equal distribution of patients lost from the experimental and control groups is only able to increase the *p* value to 0.023, failing to demonstrate a reversal of significance. FI, fragility index; LTF, lost to follow‐up; RCT, randomized controlled trials.

So, in the absence of widely agreed‐upon thresholds as to what constitutes a ‘fragile’ versus ‘robust’ RCT conclusion, how should the orthopaedic surgeon interpret fragility values, particularly as the number of studies applying the FI continues to rise? Just as a significant *p* value should not be treated as definitive evidence that a treatment effect truly exists, neither should a ‘low’ FI be treated as unshakeable evidence that an RCT's conclusions are irrelevant or prone to bias. While the number of patients lost to follow‐up is, of course, an important metric to consider when evaluating the results of an RCT, direct comparisons with the FI should be avoided, as the commonly reported claim that the results of an RCT could be flipped simply by maintaining study follow‐up is often inaccurate and misleading. In addition, a large gap has been identified between statistical methods research related to missing data and the use of these methods in application settings, including in RCTs published in the top medical journals [1]. Thus, there is also a need for improved use and reporting of appropriate methods to handle patients lost to follow‐up, including intention‐to‐treat (ITT) and multiple imputation methods [1].

It is likely that RCTs with relatively ‘low’ FIs are not as sensitive to patients lost to follow‐up as previously portrayed in the literature. Nevertheless, the demarcation between a ‘fragile’ and ‘robust’ RCT will likely remain unclear for the foreseeable future due to the sheer number of factors that contribute to the significance of a trial. Because it is difficult to account for all of these factors, including the sample size of the original trial, patients excluded, and patients lost to follow‐up, in a single metric of fragility, it will be the responsibility of the orthopaedic surgeon to holistically evaluate the significance of each RCT in its own context to determine whether a treatment effect truly exists. Thus, when interpreted in its proper context, the FI can help surgeons better assess the robustness of RCT conclusions, but it should not be the sole determinant of fragility. *p* Values, 95% confidence intervals, and FIs all have a role in assisting surgeons in determining the validity of conclusions from RCTs; however, it is unlikely that a single metric alone will be able to definitively conclude whether a trial is truly ‘fragile’ or ‘robust’.

## AUTHOR CONTRIBUTIONS

**Jacob F. Oeding**: Study design; data acquisition; data analysis; data interpretation; manuscript drafting and critical revision. **Olufemi R. Ayeni, Eric Hamrin Senorski, Stefano Zaffagnini, Alberto Grassi** and **Kristian Samuelsson**: Critical revision.

## CONFLICT OF INTEREST STATEMENT

The authors declare no conflict of interest.

## ETHICS STATEMENT

The ethics statement is not available.
